# Radiomics feature reproducibility under inter-rater variability in segmentations of CT images

**DOI:** 10.1038/s41598-020-69534-6

**Published:** 2020-07-29

**Authors:** Christoph Haarburger, Gustav Müller-Franzes, Leon Weninger, Christiane Kuhl, Daniel Truhn, Dorit Merhof

**Affiliations:** 1 Institute of Imaging and Computer Vision, RWTH Aachen University, Aachen, Germany; 2 Department of Diagnostic and Interventional Radiology, University Hospital Aachen, Aachen, Germany; 3 Fraunhofer Institute for Digital Medicine MEVIS, Bremen, Germany

**Keywords:** Cancer imaging, Tumour biomarkers

## Abstract

Identifying image features that are robust with respect to segmentation variability is a tough challenge in radiomics. So far, this problem has mainly been tackled in test–retest analyses. In this work we analyse radiomics feature reproducibility in two phases: first with manual segmentations provided by four expert readers and second with probabilistic automated segmentations using a recently developed neural network (PHiseg). We test feature reproducibility on three publicly available datasets of lung, kidney and liver lesions. We find consistent results both over manual and automated segmentations in all three datasets and show that there are subsets of radiomic features which are robust against segmentation variability and other radiomic features which are prone to poor reproducibility under differing segmentations. By providing a detailed analysis of robustness of the most common radiomics features across several datasets, we envision that more reliable and reproducible radiomic models can be built in the future based on this work.

## Introduction

Radiomic image analysis aims at extracting mineable, quantitative features from medical images. Based on this data, quantitative models for classification, prediction, prognostication and treatment response may be built. To this end, a single entity such as a tumour, is characterized by a set of image features that constitute the entity’s radiomic signature. In the recent past, numerous radiomic signatures have been developed, that hold promise for clinical application^1–3^.

However, the introduction of radiomics into clinical practice has been lacking. This is in large parts due to the difficulties in reproducibly extracting radiomic features and the resulting variability^4^. In the chain between image acquisition and extraction of radiomic features, a multitude of parameters may influence radiomics features: First, the choice of image acquisition parameters and scanner site as examined by Berenguer et al.^5^ and Peerlings et al.^6^. Second, reconstruction algorithms such as filtered back projection or iterative reconstruction, whose influence has been examined by several research groups recently^7–9^. Third, the choice of software to extract the radiomic features has a significant influence. This problem has recently been tackled by the Image Biomarker Standardization Initiative^10^. Finally, the tumour has to be segmented, which is mostly performed manually by medical experts. Although this last part is probably the most obvious source of variability between readers and is often recognized as a source of potential problems in areas outside of radiomics^11^, it has not yet been comprehensively examined in radiomics—most likely due to the difficulties in building a sufficiently large dataset of tumours labelled by several raters.

Thus, it has been a largely unanswered question to what degree segmentation variability has an impact on radiomics features. We therefore set out to analyse this influence and to work out, which radiomic features are stable under varying segmentations as typically encountered in the clinics.

### Related work

Kalpathy-Cramer et al.^3^ have assessed the variability of radiomics features to variations in the segmentation for lung nodules based on automated segmentation and varying feature implementations. In^12^, Balaguranathan et al. have performed a similar analysis, building an ensemble of a manual and automated segmentation approach. Parmer et al.^13^ found that features extracted from automatic segmentations had a better reproducibility that those extracted from manual segmentations. Tixier et al.^14^ have investigated segmentation variability between two raters and manual and semi-automatic segmentation methods for MR images of glioblastoma. They found that variation between two consecutive scans was higher than variation between segmentations for most features. Qiu et al.^15^ compared feature reproducibility across five manual segmentations as well as a semiautomatic approach and found that about 50% of radiomics features showed strong robustness with respect to segmentation variability. Zwanenburg et al.^16^ assessed radiomics feature robustness by image perturbation in computed tomography (CT) images. Yamashita et al.^17^ found that for contrast-enhanced CT images of patients with pancreatic cancer, scan parameters had stronger influence on radiomics features than segmentation variability. Tunali et al.^18^ assessed reproducibility of radiomic features extracted from peritumoral regions of lung cancer lesions. In a comprehensive feature analysis of head and neck squamous cell carcinoma, pleural mesothelioma and non-small cell lung cancer lesions based on three expert segmentations, Pavic et al.^19^ found that inter-rater variability has a significant influence on radiomics features.

While all these works aim at assessing feature reproducibility with respect to segmentation variability, the segmentations based on which the analyses were carried out originate from only two raters at most. However, it has been shown recently by Joskowicz et al. that inter-rater variability in segmentation of lesions in CT images cannot be adequately captured by two raters only^20^. In fact, it was found that more than three raters are required in order to capture the full distribution of plausible segmentations. Since manual segmentation of large-scale datasets by multiple raters is practically infeasible even in research settings, an alternative solution has been proposed in Haarburger et al.^21^. Here, the authors automatically generated 25 plausible segmentations using a probabilistic U-Net^22^. Based on these, they analysed feature repeatability with respect to segmentation variability and identified groups of features that are more or less stable. However, the Probabilistic U-Net suffers from limited segmentation diversity^21, 23^. Moreover, the evaluation was carried out on a single dataset only. Several extensions and modifications of the Probabilistic U-Net have been published recently: Hu et al.^24^ introduced variational dropout^25^ after the last convolutional layer of the U-Net to estimate epistemic uncertainty in the produced segmentations. In^26^, the original authors of the Probabilistic U-Net improved their work by proposing a hierarchical latent space decomposition, which aimed at improving segmentation diversity by modelling the segmentation distribution at various scales. The same idea was simultaneously proposed as PHiSeg by Baumgartner et al.^23^.

### Contributions

In this work, we comprehensively evaluate, how differences in outlining the tumour on CT images result in variability in radiomic features. In particular, we examine which features are unstable towards this unavoidable uncertainty in tumour outlines and should be regarded with care in future studies. To this end, we proceed in two steps: first, we employ a CT image dataset with lung nodules which were each outlined by four human readers and investigate the resulting variations in radiomic features by quantifying human inter-reader variability. Second, we make use of a convolutional neural network to both generate an even greater number of segmentations (n = 34.400) and extend our analysis to additional datasets: Building on PHiSeg^23^, we generate plausible and diverse segmentations for three publicly available radiological datasets of lung nodules (LIDC challenge dataset), liver tumours (LiTS challenge dataset) and kidney tumours (KiTS challenge dataset). We analyse feature reproducibility with respect to the segmentation distribution provided by PHiSeg on all three datasets. In a comprehensive analysis we compare feature reproducibility both across these three datasets and between human and machine labelled segmentations and identify features that are consistently stable or unstable, respectively. We believe that excluding features that we identified as consistently unstable from radiomic analyses will improve reproducibility of radiomics signatures for clinical applications in the future.

## Methods

### Image data

We assessed feature robustness on three datasets as shown in Table 1:

1. The public Lung Image Database Consortium (LIDC-IDR) dataset^27, 28^ consisting of 1035 helical thoracic CT images and including manual lung lesion segmentations from four expert raters. These scans originate from seven academic institutions covering scanner models from four different vendors.
2. The Kidney Tumour Segmentation Challenge (KiTS) dataset^29^ containing 300 CT images from the late arterial phase of kidney tumours. This dataset orignates from single institution and includes a single lesion segmentation mask per scan, provided by an expert.
3. The Liver tumour Segmentation Challenge (LiTS) dataset^30^ consisting of CT images of 201 patients with liver tumours and a single lesion segmentation mask provided by an expert. The data originates from seven institutions.

Informed consent was obtained from all patients.

### Probabilistic segmentation

Our workflow follows the core steps described in Haarburger et al.^21^. In order to generate the automatic segmentations, we build on the PHiSeg neural network architecture^23^, which incorporates a U-Net^31^ and a variational autoencoder (VAE) as proposed by Kohl et al.^22^. Given an input image, plausible segmentations of a tumour are generated by the neural network. The segmentations mimic those provided by human readers. The original Probabilistic U-Net suffered from limited segmentation diversity^21, 23^. To overcome these limitations, in PHiSeg, the latent space in the VAE part of the network is decomposed into several scales. Deep supervision is added at each resolution level during training using a binary cross entropy loss function.

For each of the three datasets, PHiSeg is trained separately using default parameters as provided in the reference implementation. We split the data into training, validation and test set as provided in the LIDC dataset to optimize hyperparameters. For the KiTS and LiTS datasets, hyperparameters were set as in LIDC. Therefore, for these two datasets a split into training and test set is sufficient. Since a segmentation ground truth for these two datasets is only available for the challenge training set, the split into our training and test sets is based on this publicly available training set in an (80%/20%) ratio. An overview of the splits per dataset is provided in Table 1.

**Table 1:** Number of available datasets and their respective split into training, validation and test-data for each of the three datasets utilized in this work.

	Training	Validation	Test
LIDC	560	140	175
KiTS	168	–	42
LiTS	105	–	26

**Figure 1:**
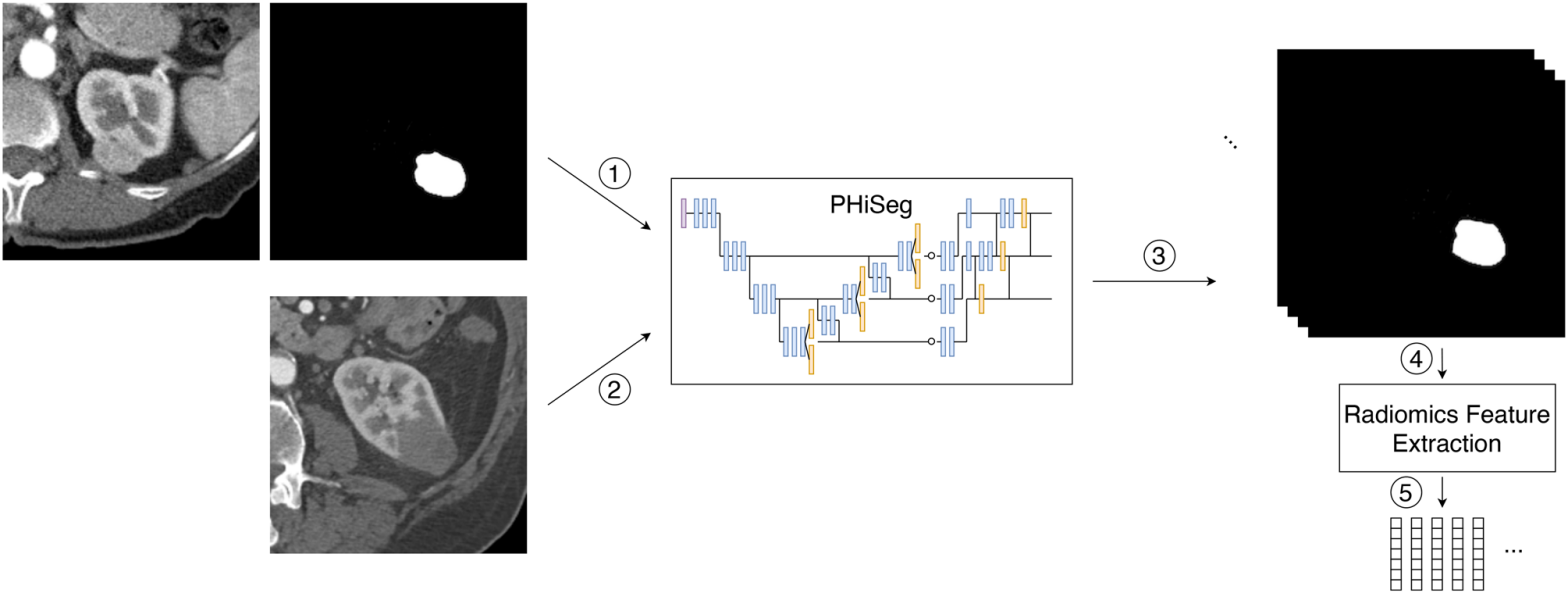
The following pipeline is set up for each of the three datasets: (1) The PHiSeg network is trained using CT images and corresponding expert annotations. After training, given an unseen tumour image (2), the network samples ($N =25$) possible segmentations for that image (3). Based on these segmentations for a single tumour, ($N = 25$) possible radiomics feature vectors are extracted (4). Finally, feature variability across the possible segmentations is calculated (5).

An overview of our workflow is depicted in Fig. 1. Each dataset is first split into training, validation (only for LIDC) and test set. Then, PHiSeg is trained and optimized using the training and validation data (1) for which both images and expert segmentations are utilized. After training, unseen test images are fed into the trained network (2) and 25 plausible segmentations are sampled for each given tumour (3). For each segmentation mask, statistics, shape and texture features are extracted (4) resulting in ($N=25$) radiomic feature vectors for each given tumour (5). Finally, feature variability across segmentations is calculated using ICC.

For each dataset we cropped the images to the region of interest, i.e. slices of the LIDC dataset were cropped to ($128 \times 128$) voxels and for KiTS and LiTS we cropped a ($192 \times 192$) around the lesion centre, which provides sufficient context for lesion segmentation (see Fig. 5 for examples). The larger crop for LiTS was chosen because the liver has a larger extent in the axial plane than kidney and lung lesions. The lesion centre was defined as the center of a rectangle with a minimum size such that the whole lesion is covered by that rectangle. Moreover, for KiTS and LiTS we masked all images with the kidney and liver binary mask, respectively, provided in the respective dataset. This prevents the network to learn spurious correlations from locations outside of the organ in question. As PHiSeg operates in 2D, we train on all axial slices containing a lesion. For testing, we picked for each lesion the slice that contained the largest segmented area in the ground truth and sampled 25 segmentations from this principal axial slice.

During the sampling process, a sample was only accepted if the Dice coefficient between the PHiSeg segmentation and expert segmentation (LIDC: any of the expert segmentations) was > 0.3. This particular threshold was chosen based on the histogram of all pairwise Dice scores in the LIDC training set, which is included in Fig. 7 the supplementary material. Moreover, the minimum volume for a lesion to be considered was set to $30\,\mathrm {mm}^3$, which corresponds to a radius of $1.92\,\mathrm {mm}$ for a sphere. In this way, we made sure that the features that are extracted in the next step relate to the same region in the image. Moreover, for lesions that are very small in comparison to the voxel geometry, partial volume effect would have a very strong impact on the resulting voxel intensities and extracted radiomics features. For the purpose of radiomics feature repeatability assessment, we neglected slices with several, but distinct segmentations that related to the same lesion but were connected on another slice. This prevented incorrect feature calculations for shape features that are only comparable when extracted on single interconnected objects. As a side note, this condition only applied in less than 0.5% of the image data.

### Assessment of automated segmentation quality

**Figure 2:**
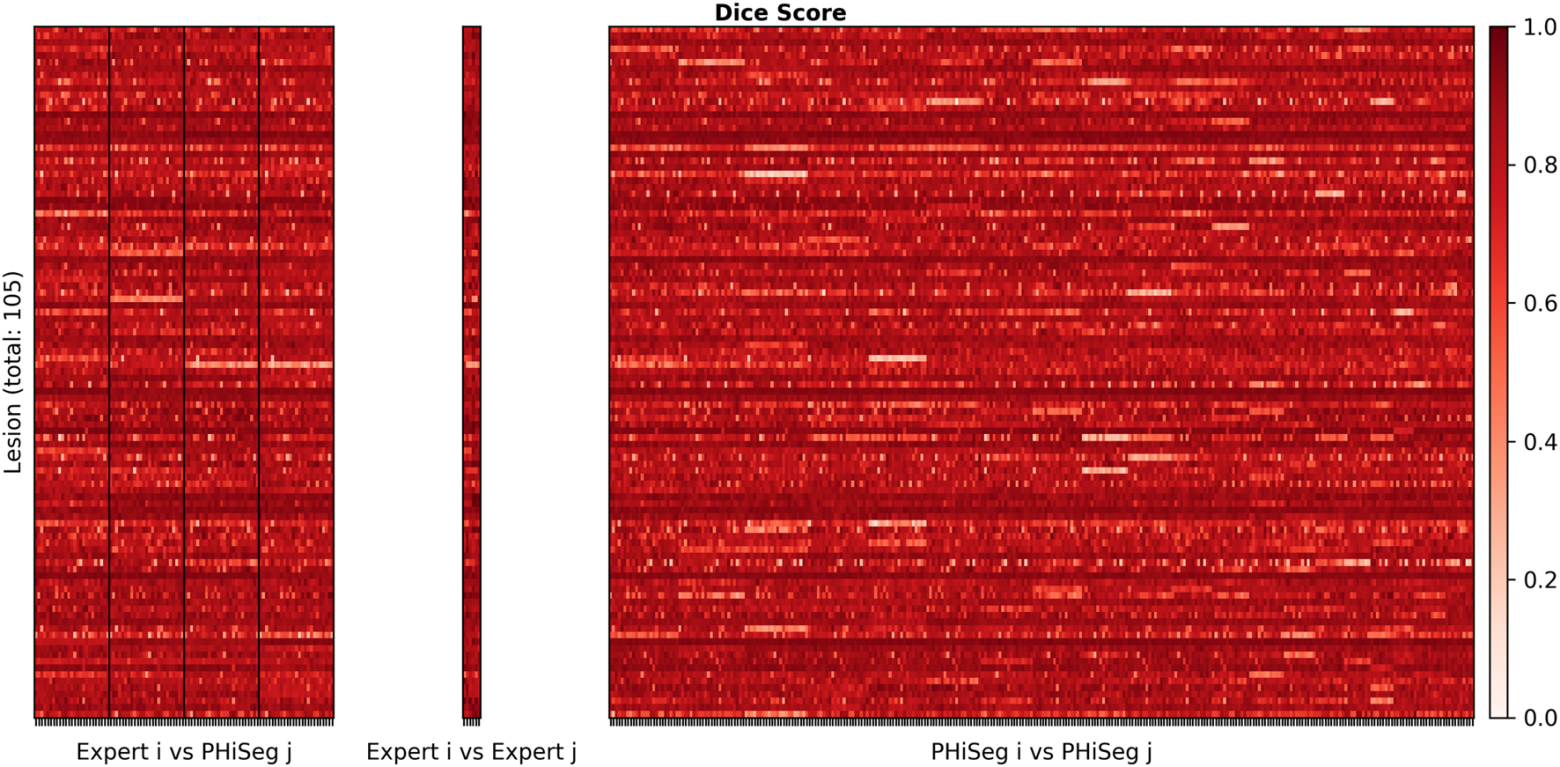
Pairwise Dice scores between expert raters and between PHiSeg segmentations (left), expert raters (middle) and between PHiSeg segmentations (right) for the LIDC dataset.

Only if the automatically generated segmentations are sufficiently realistic and accurate, feature robustness analysis is meaningful and valid. Therefore, before assessing feature robustness, we evaluated segmentation quality produced by PHiSeg. To this end, on the LIDC dataset we evaluated the Dice score pairwise between expert raters and PHiSeg, between expert raters and between PHiSeg “raters” (Fig. 2).

### Feature extraction

For image preprocessing, all images were resampled to $1 \times 1 \times 1 \,\mathrm {mm}^3$. This is necessary to compare features scores across several datasets that were acquired by several CT scannerse. If the voxel size is not resampled to a common spacing, extracted features do not refer to the same physical space across scanners and are thus not comparable. Moreover, we binned all grey values using a bin width of 25 HU. We employed PyRadiomics^32^ as an open source implementation for extraction of radiomics features. In total, 92 radiomics features were extracted:

- 18 statistics features
- 12 shape features
- 22 gray level co-occurence matrix (GLCM) features
- 16 gray level size zone matrix (GLSZM) features
- 16 gray level run length matrix (GLRLM) features
- 5 neighbourhood difference gray tone matrix (NDGTM) features

The features were not standardized or scaled before further subsequent analyses.

### Inter-reader agreement

In order to assess feature robustness across segmentations, we evaluated the intraclass correlation coefficient $\mathrm {ICC}(1,1)$^33, 34^ based on a one-way random model. This definition of ICC assumes no systematic bias and has been used previously in radiomics feature reliability studies^35^. In essence, the ICC quantifies inter-rater variability with a value of one indicating perfect agreement between raters on a radiomic feature for a specific tumour and a value of zero indicating complete randomness. We evaluated the ICC both for the human readers on the LIDC dataset and for the automatically generated segmentations on all three datasets.

## Results

### Agreement between automated and manual segmentations

Dice scores between expert readers pairwise as well as between expert readers and PHiSeg segmentations denote a high overlap with a median Dice score of $0.87\ \mathrm {IQR}\ [0.8\ 0.91]$ between expert readers and $0.85\ \mathrm {IQR}\ [0.77\ 0.89]$ between PHiSeg segmentations and expert readers. Examples for segmentations as provided by the automated method (PHiSeg) versus the ground truth segmentation(s) as provided by expert human readers on all three datasets are provided in Fig. 5.

### Radiomics feature reproducibility

**Figure 3:**
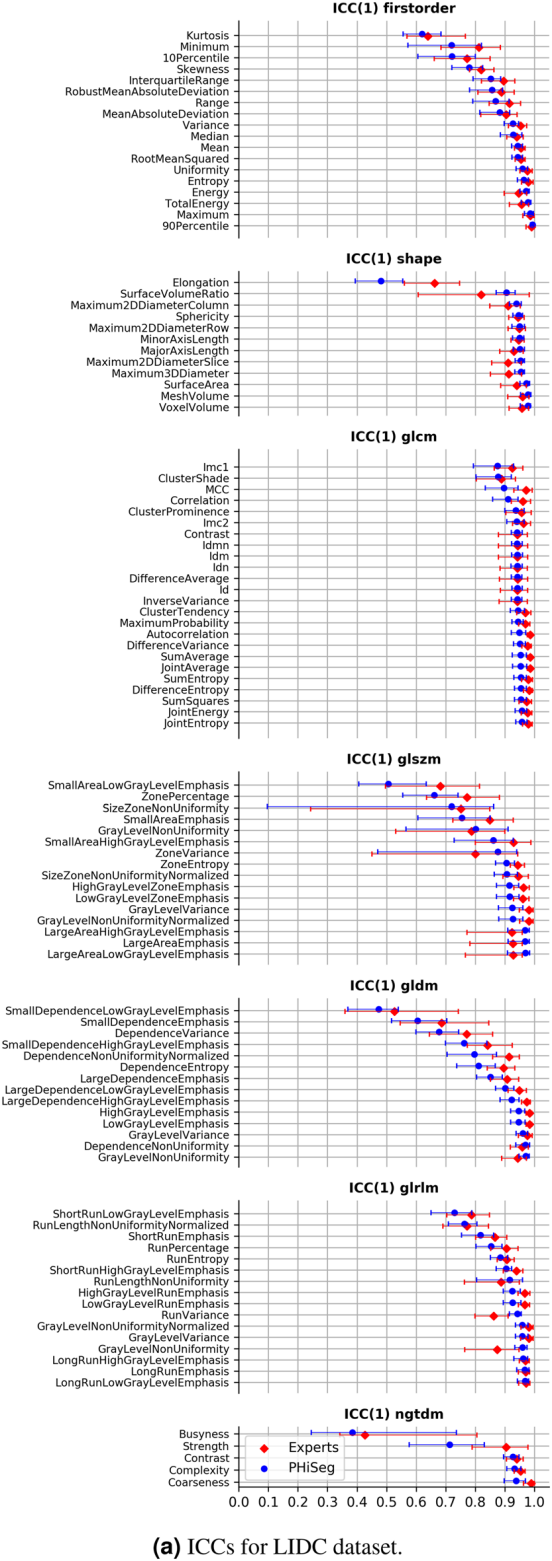
ICC on LIDC dataset between individual features and PHiSeg raters (blue) and expert raters (red) grouped by feature category and sorted by ICC.

**Figure 4:**
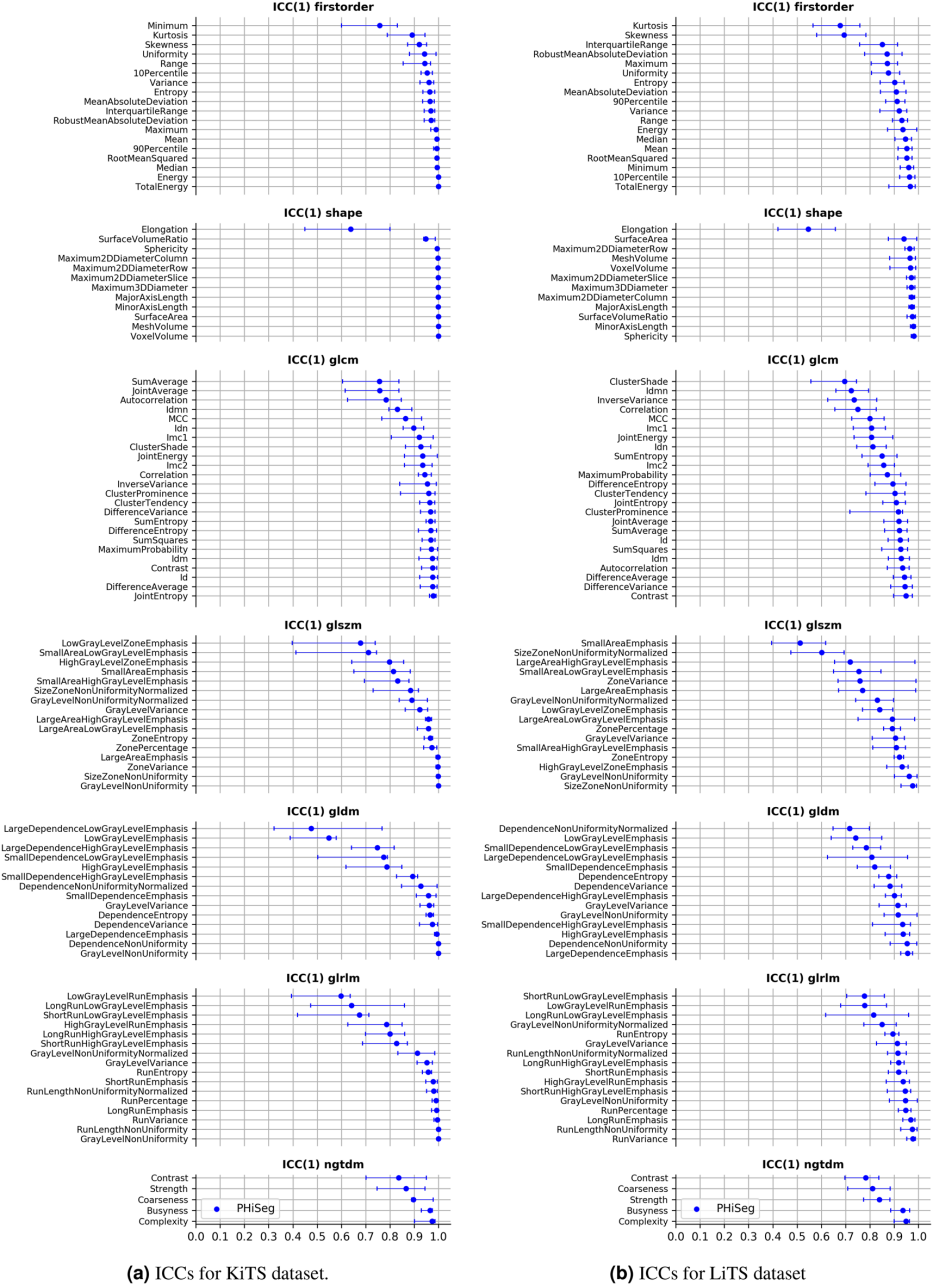
ICC between individual features and PHiSeg raters for KiTS (**a**) and LiTS (**b**) datasets grouped by feature category and sorted by ICC.

Figure 3a illustrates ICCs for the LIDC dataset both between the four expert readers (red) and between the 25 segmentations provided by the automated method (blue), while Fig. 4a and b show the ICCs based on the automated segmentations for liver and kidney tumours. A comprehensive overview over all ICCs for all radiomic features and each dataset is given in 2. The 95% confidence intervals were calculated using 1000 bootstrap iterations. We found that the ICCs based on the two types of segmentation approaches (human vs. automated) were highly correlated with a Pearson correlation coefficient of $r = 0.921$. In general, features that were found to be unstable based on human annotations were also found to be unstable based on automated annotations. Irrespective of feature categories, most features (84% and 88% for PHiSeg an expert raters) exhibited an ICC> 0.8. Overall, the highest ICCs were achieved for shape and first order features.

Consistent results were found across all tumour categories and segmentation methods: when features exhibited high ICCs (i.e. ICC > 0.9) on one dataset they also achieved high ICCs on the other datasets. This consistency is strong in particular for shape, first order and glcm features, with mean ICCs of 0.93, 0.91 and, 0.92, respectively. Figure 6 illustrates the mean ICCs over all feature categories. It is of particular interest in this regard, that the arguably most clinically used shape feature—the maximum tumour diameter on a 2D slice—exhibits an ICC of 0.91 among human readers in the LIDC dataset, which is comparatively low as compared to the ICC of the otherwise mostly highly consistent shape features.

**Figure 5:**
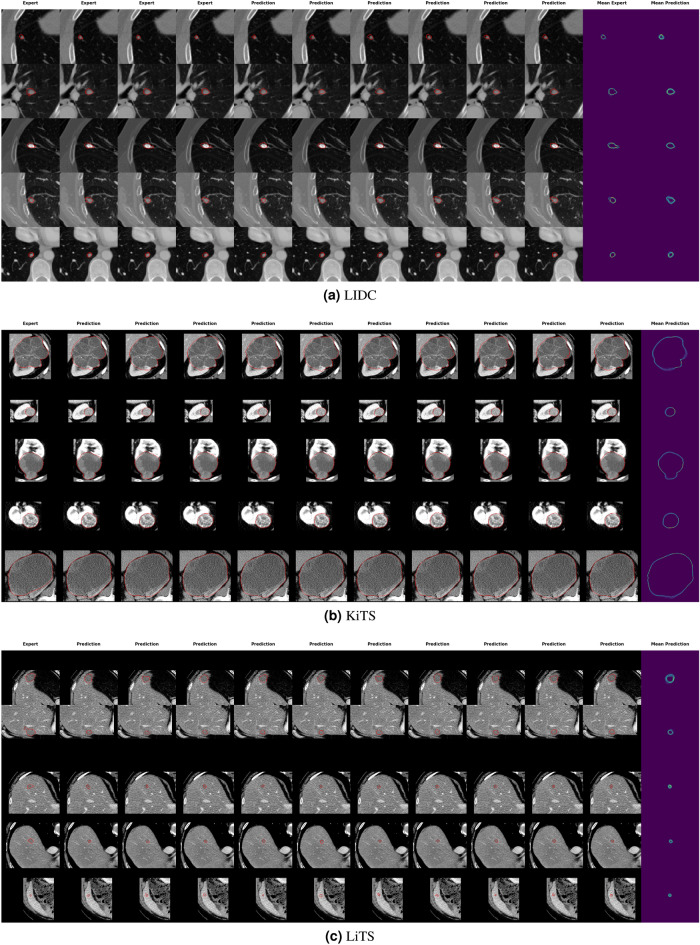
Examples for PHiSeg segmentations on LIDC (**a**), KiTS (**b**) and LiTS (**c**) datasets. Aggregations of all 25 segmentations generated by the neural network are denoted in the rightmost column, respectively. Note that four expert segmentations are only available for the LIDC dataset, while the other datasets only contain one expert segmentation each.

**Figure 6:**
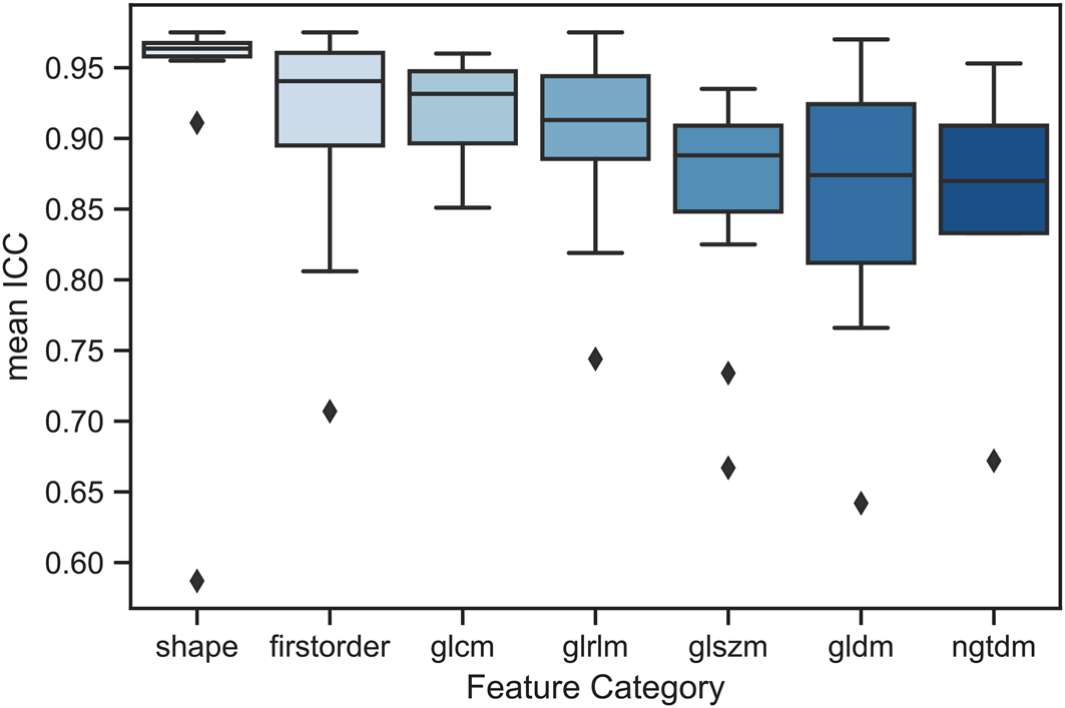
Mean ICC across all three datasets by feature categories.

## Discussion

Despite its promise of advancing personalized medicine and supporting radiologists in diagnostic and clinical decisions, the implementation of radiomic analysis in clinical routine is still missing^1–3^. The most likely reasons for this lack of clinical translation are difficulties in reproducibly extracting radiomic features from images^4^. The potential sources of variability have been identified previously and of the four main influences, three have already been examined extensively: image acquisition parameters^5, 6^ reconstruction algorithms^7–9^ and differences in the software framework^10^. However, a large scale evaluation of the confounding effects of segmentations by different readers have been missing so far. Thus, the aim of this study was to investigate the impact of segmentation variance on radiomics features in three large publicly available datasets of CT images. We used manual segmentations by human experts on a dataset of lung nodules in CT images to assess inter-reader variability. To further analyse radiomic feature reproducibility on a dataset of liver and kidney tumours and to broaden the data basis, we employed a probabilistic segmentation algorithm to generate a multitude of realistic segmentations for each tumour in all three datasets. To this end, we generated plausible segmentations (N = 25 for each lesion) by PHiSeg and computed the full set of radiomics features for all segmentations. Our analysis was performed on three public segmentation challenge datasets: lung nodule segmentation (LIDC), kidney tumour segmentation (KiTS) and liver tumour segmentation (LiTS).

As depicted for the LIDC dataset in Fig. 2, the pairwise Dice scores between expert raters (a) and pairwise Dice scores between expert raters and PHiSeg segmentations are in a comparable range for most lesions in the dataset. This indicates that PHiSeg produces segmentations that are plausible and mimic the variations between several experts realistically. This finding can also be observed qualitatively in Fig. 5 for LIDC, KiTS and LiTS datasets. More examples are provided in the supplementary material. The same conclusion was drawn in Baumgartner et al.^23^, where PHiSeg performance was compared with a probabilistic U-Net^22^, resulting in a performance that was on par with a deterministic U-Net^31^. We thus are confident that PHiSeg segmentation accuracy was sufficient to support a valid analysis on extracted radiomics features.

As a measure of inter-reader agreement, we made use of the ICC. As an alternative, we could have chosen overall concordance correlation coefficient (OCCC)? which is used in other similar studies. However, we concluded from^35^ that most other studies on radiomics reproducibility used ICC. In order to maintain comparability of our results with the majority of other works, we decided to use ICC. A cut-off ICC value ensuring reproducible features has not yet been established. Possible choices are the often-used interpretation of defining excellent agreement as ICC > 0.75^36^ or the interpretation proposed by Koo and Li^37^, stating that ICC > 0.9 corresponds to excellent agreement. Zwanenburg et al.^16^ have adopted the rather conservative categorization in Koo and Li^37^.

In our analysis, the ICCs based on experts and PHiSeg were highly correlated, indicating that PHiSeg generated segmentations are comparable to manual segmentations by experts. In our analysis of radiomic feature reproducibility, we found consistent results over all datasets: Individual features that exhibited a high ICC on one dataset were similarly robust on the others, whereas features with low ICCs were unstable on the other dataset as well. We were thus able to show that there are subsets of radiomics features that are consistently highly robust and others that are highly sensitive with respect to segmentation variability across datasets. Feature reproducibility differed between feature categories. As indicated in Fig. 6, shape features were best reproducible overall, followed by firstorder and glcm. This means that features quantifying texture tended to be of worse reproducibility than shape. One possible reason might be changes with respect to where or how to define the exact contour of a lesion: Especially for the lung dataset, the intensity difference between lesion and background (air) is very high, so if “air voxels” are included in the contour, this has a strong impact on many non-shape features.

Zwanenburg et al.^16^ have reported comparable ICCs of non-small-cell lung cancer CT images under image perturbations. On a head-and-neck squamous cell carcinoma CT dataset, reported ICCs were generally lower. Kalpathy-Cramer et al.^3^ have reported that 68% of features were reproducible across segmentations with a concordance correlation coefficient of > 0.75. In Haarburger et al.^21^, a similar analysis was carried out on a lung cancer dataset using a probabilistic U-Net^22^. It was shown that in every feature category there are features that are stable and poorly stable across segmentations, respectively. However, the method in Haarburger et al.^21^ suffered from limited segmentation diversity which was overcome by PHiSeg as shown in Baumgartner et al.^23^.

Based on our findings we envision the following implications for radiomic signature development. Rather than performing a “standard” feature selection, the curse of dimensionality could be considerably alleviated by focusing on robust features only and neglecting features that we have proven to be consistently prone to poor repeatability across datasets.

Our work has several limitations: Our analysis is based on CT images. Future research has to determine, if our findings apply to e.g. MRI or PET images. Moreover, among the many confounding effects in radiomics such as scanner device, vendor, reconstruction method, image preprocessing and feature implementation, we only examined the influence of segmentation variability. Yamashita et al. claimed that variations between scans had a higher impact on reproducibility than segmentation^17^. An additional aspect that was not covered in this work is the question as to what extent feature reproducibility translates into the reproducibility of a whole radiomic signature, i.e. when several features are combined in model. It should also be noted, that our analysis was solely based on 2D axial slices rather than 3D volumetric segmentations. This is due to the large memory consumption of PHiSeg, which makes an extension to 3D infeasible for current graphics cards. However in our clinical experience, many segmentation tasks are carried out slicewise in 2D. Future work should extend the analysis to volumetric probabilistic segmentations, though. Moreover, we disregarded slices with disconnected lesions that belonged to the same lesion entity but were connected on another slice. In the 2D case, an inclusion of such slices would heavily affect radiomics shape features such as surface-to-volume ratio, major-axis length. This limitation could also be overcome in the future by using 3D segmentations.

## Conclusions

Using a set of manual and automated plausible segmentations, we analysed variance of radiomic features in three CT datasets of lung, liver and kidney tumours and found consistent results by identifying groups of image features that are subject to different degrees of robustness, even across datasets. These findings can be used in future studies by building radiomic models based on features that we identified as being robust with respect to segmentation variability. We envision that this approach helps in producing more reproducible and more widely applicable radiomic models.

## Supplementary information

Supplementary Information.

## Data Availability

The three datasets used in this work are publicly available: The public Lung Image Database Consortium (LIDC-IDR) dataset is available at TCIA^27, 28, 38^. The KiTS dataset^29^ is available on GitHub: https://github.com/neheller/kits19 and the LiTS dataset^30^ is available on CodaLab: https://competitions.codalab.org/competitions/17094. We utilize the publicly available implementation of PHiSeghttps://github.com/baumgach/PHiSeg-code.
