# Author Correction: Radiomics feature reproducibility under inter-rater variability in segmentations of CT images

**DOI:** 10.1038/s41598-021-02114-4

**Published:** 2021-11-16

**Authors:** Christoph Haarburger, Gustav Müller‑Franzes, Leon Weninger, Christiane Kuhl, Daniel Truhn, Dorit Merhof

**Affiliations:** 1 Institute of Imaging and Computer Vision, RWTH Aachen University, Aachen, Germany; 2 Department of Diagnostic and Interventional Radiology, University Hospital Aachen, Aachen, Germany; 3 Fraunhofer Institute for Digital Medicine MEVIS, Bremen, Germany

Correction to: *Scientific Reports* 10.1038/s41598-020-69534-6, published online 29 July 2020

The original version of this Article contained errors in the Author Contributions section,

“C.H. and D.T. conceived the experiments, G.M.-F. conducted the experiments, C.H., D.T., G.M.-F., D.M., C.K. and L.W. analysed the results. C.H. and D.T. wrote the manuscript. All authors reviewed the manuscript.”

now reads:

“C.H. and D.T. conceived the experiments, C.H. and G.M.-F. conducted the experiments, C.H., D.T., G.M.-F., D.M., C.K. and L.W. analysed the results. C.H. wrote the manuscript. All authors reviewed the manuscript.”

The original Article has been corrected.

