# Supplementary material for: Radiomics feature reproducibility under inter-rater variability in segmentations of CT images
Source: Sci Rep. 2020 Jul 29;10:12688. doi: 10.1038/s41598-020-69534-6 (PMC7391354; doi:10.1038/s41598-020-69534-6)
Supplement: Supplementary file 1 — Supplementary Information. [file 41598_2020_69534_MOESM1_ESM.pdf]

Radiomics Feature Reproducibility Under Inter-Rater Variability in Segmentations of CT Images

Christoph Haarburger, Gustav Müller-Franzes, Leon Weninger, Christiane Kuhl, Daniel Truhn, Dorit Merhof

Supplementary Information

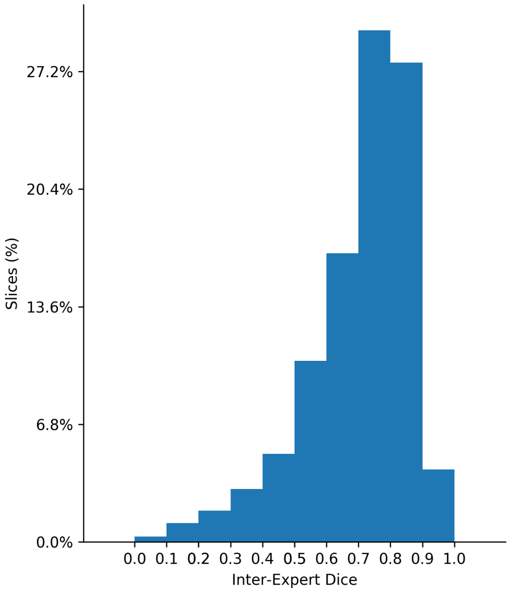

Figure 7. Pairwise 2D Dice scores of all raters in the LIDC training set.

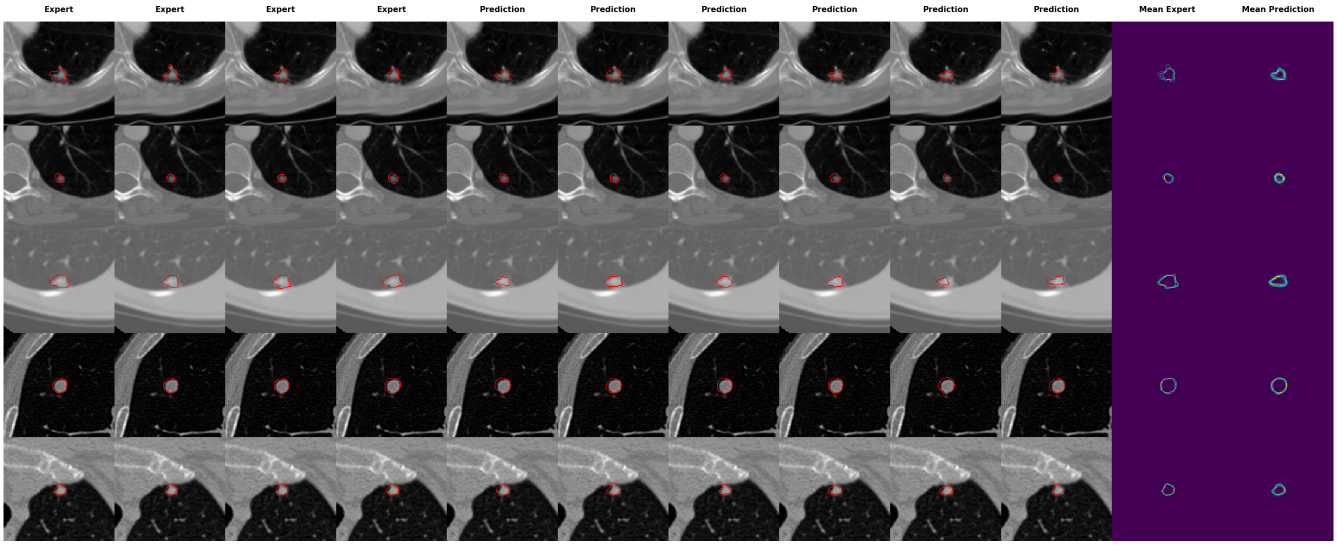

(a) Sample 1

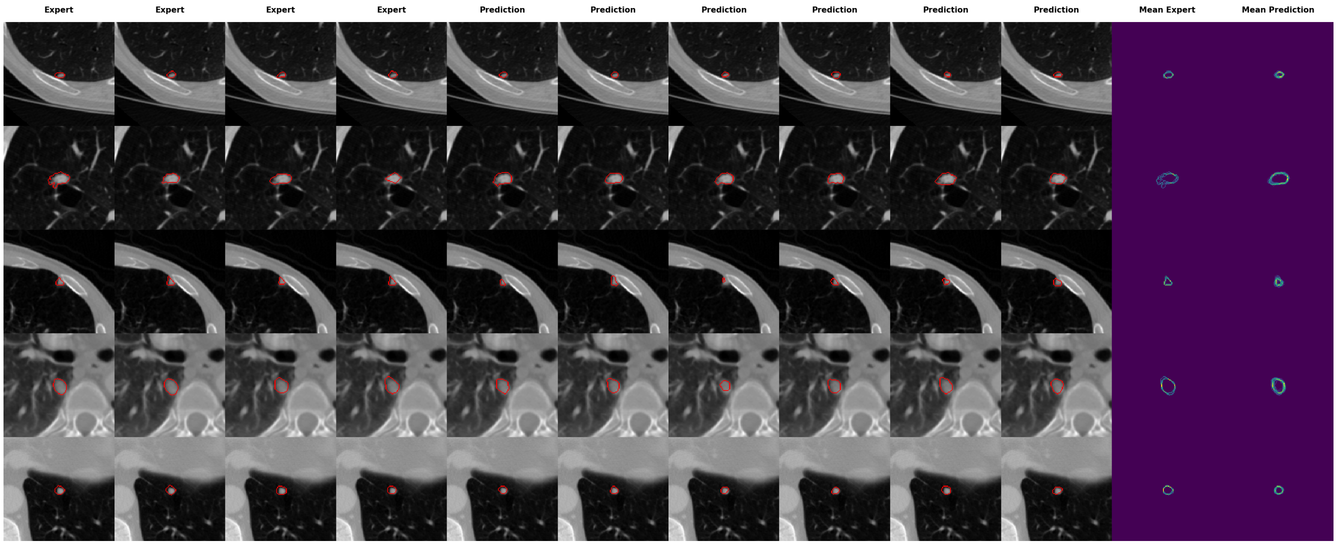

(b) Sample 2

Figure 8. Segmentation examples for LIDC dataset

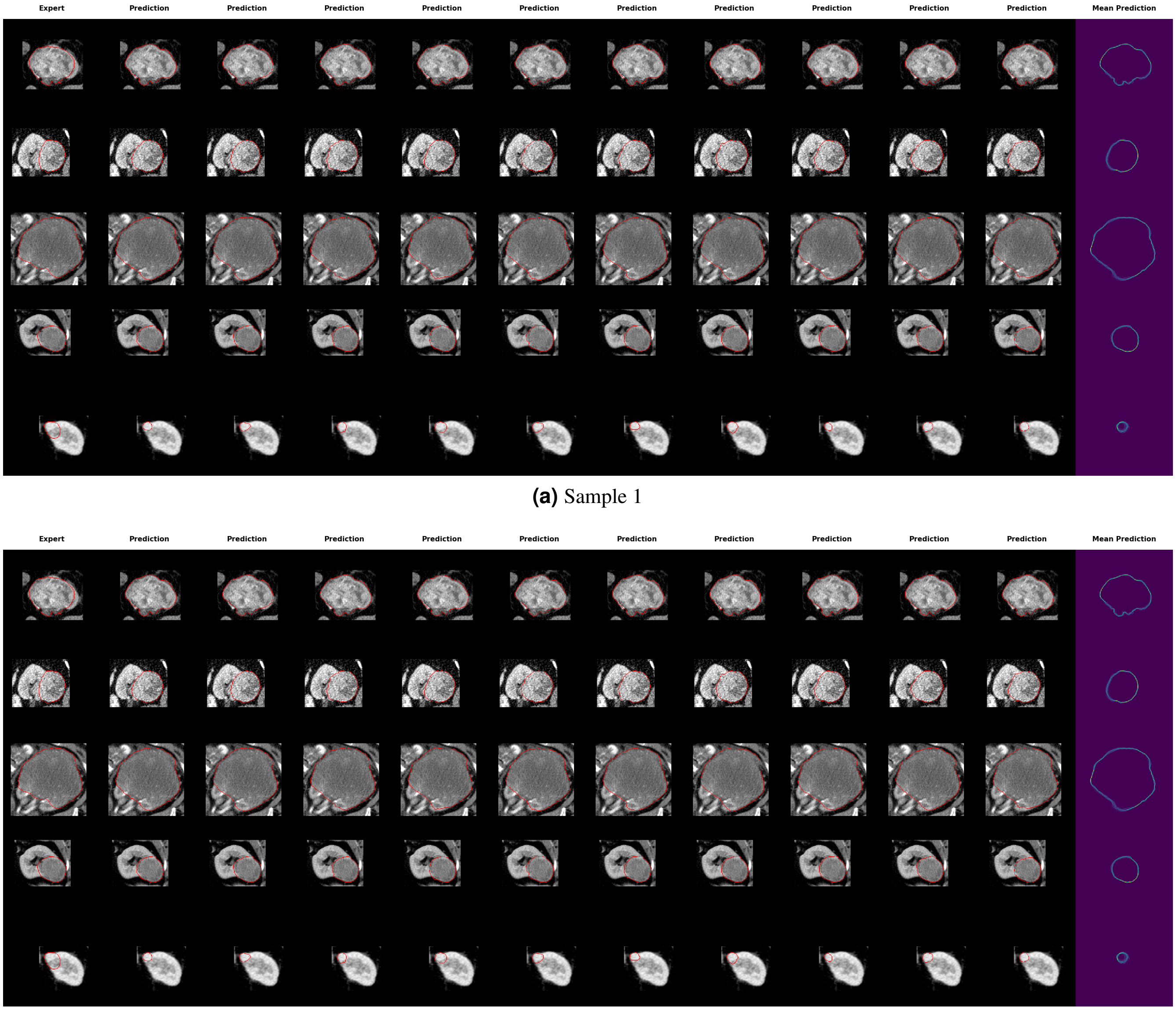

(a) Sample 1

(b) Sample 2

Figure 9. Segmentation examples for KiTS dataset

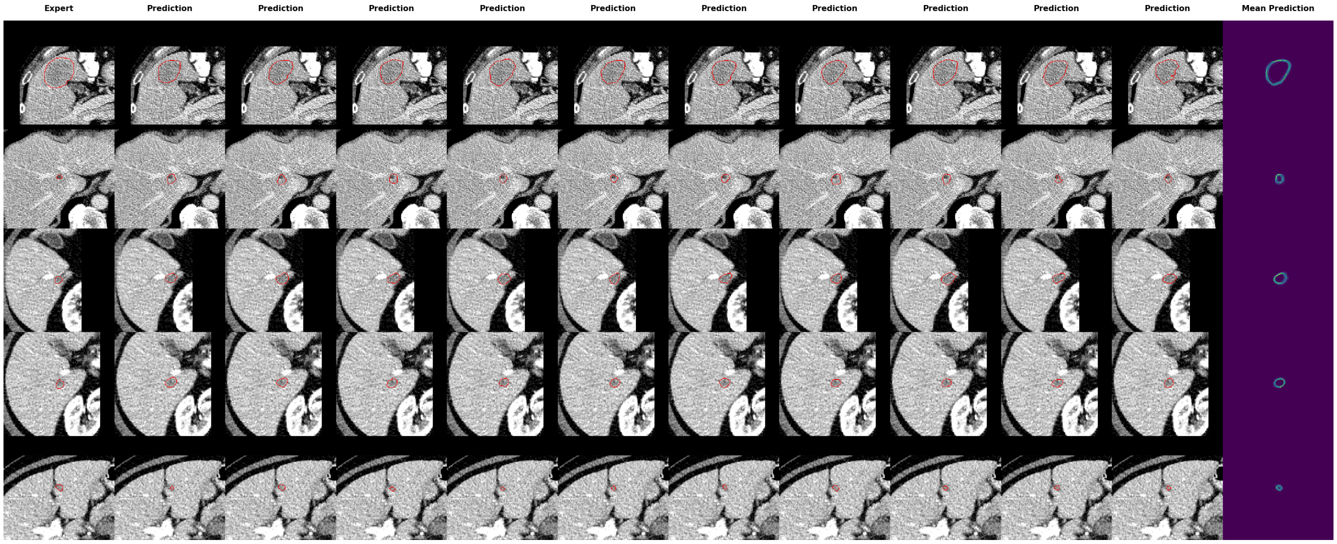

(a) Sample 1

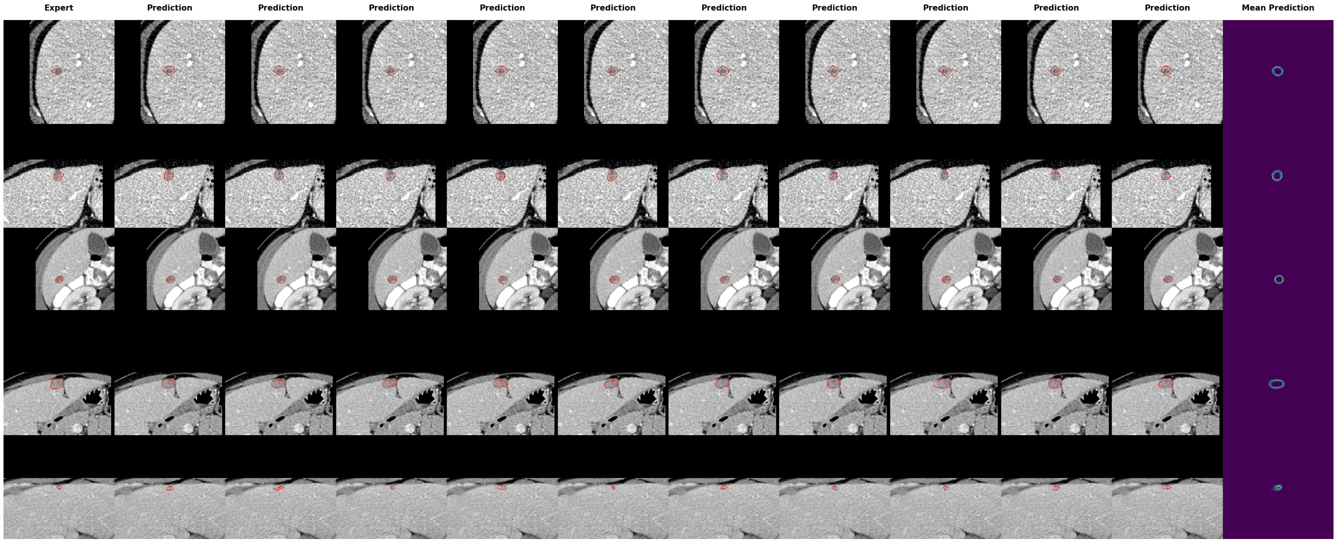

(b) Sample 2

Figure 10. Segmentation examples for LiTS dataset

	LIDC Experts	LIDC PHiSeg	LiTS PHiSeg	KiTS PHiSeg	mean_ICC
original_shape_MeshVolume	0.959	0.978	0.965	1.0	0.975
original_shape_VoxelVolume	0.957	0.978	0.966	1.0	0.975
original_firstorder_TotalEnergy	0.957	0.978	0.966	1.0	0.975
original_glrlm_LongRunEmphasis	0.971	0.968	0.968	0.992	0.975
original_firstorder_90Percentile	0.989	0.994	0.914	0.993	0.972
original_gldm_DependenceNonUniformity	0.96	0.971	0.951	1.0	0.97
original_shape_MinorAxisLength	0.947	0.952	0.979	0.999	0.969
original_shape_Sphericity	0.945	0.947	0.982	0.995	0.967
original_shape_Maximum2DDiameterRow	0.948	0.951	0.964	0.998	0.965
original_shape_MajorAxisLength	0.931	0.951	0.973	0.999	0.964
original_firstorder_Energy	0.946	0.972	0.934	1.0	0.963
original_shape_SurfaceArea	0.94	0.973	0.939	1.0	0.963
original_firstorder_Mean	0.955	0.944	0.953	0.993	0.961
original_firstorder_RootMeanSquared	0.955	0.944	0.953	0.994	0.961
original_glcm_DifferenceVariance	0.977	0.951	0.945	0.968	0.96
original_firstorder_Maximum	0.985	0.986	0.875	0.99	0.959
original_shape_Maximum3DDiameter	0.911	0.955	0.971	0.999	0.959
original_shape_Maximum2DDiameterSlice	0.911	0.955	0.971	0.999	0.959
original_glcm_SumSquares	0.974	0.954	0.93	0.969	0.957
original_glcm_JointEntropy	0.979	0.957	0.91	0.979	0.956
original_gldm_GrayLevelNonUniformity	0.942	0.971	0.911	1.0	0.956
original_shape_Maximum2DDiameterColumn	0.91	0.939	0.973	0.998	0.955
original_gldm_GrayLevelVariance	0.976	0.96	0.92	0.962	0.955
original_glrlm_GrayLevelVariance	0.98	0.959	0.918	0.955	0.953
original_ngtdm_Complexity	0.953	0.932	0.95	0.975	0.953
original_firstorder_Entropy	0.979	0.964	0.902	0.963	0.952
original_firstorder_Median	0.939	0.927	0.947	0.995	0.952
original_glcm_Contrast	0.94	0.942	0.949	0.974	0.951
original_glcm_DifferenceEntropy	0.982	0.955	0.895	0.967	0.95
original_glcm_DifferenceAverage	0.94	0.942	0.94	0.974	0.949
original_glcm_ClusterTendency	0.969	0.945	0.907	0.966	0.947
original_glrlm_RunLengthNonUniformity	0.893	0.92	0.973	1.0	0.947
original_glcm_Idm	0.94	0.942	0.929	0.974	0.946
original_glcm_Id	0.94	0.942	0.924	0.974	0.945
original_glrlm_RunVariance	0.862	0.942	0.977	0.996	0.944
original_glrlm_GrayLevelNonUniformity	0.872	0.961	0.943	1.0	0.944
original_glcm_ClusterProminence	0.955	0.939	0.919	0.962	0.944
original_firstorder_Variance	0.954	0.928	0.923	0.963	0.942
original_glcm_MaximumProbability	0.969	0.945	0.872	0.969	0.939
original_firstorder_Uniformity	0.976	0.96	0.877	0.943	0.939
original_glcm_SumEntropy	0.979	0.954	0.853	0.968	0.939
original_glszm_ZoneEntropy	0.944	0.906	0.923	0.968	0.935
original_glszm_GrayLevelVariance	0.981	0.925	0.907	0.926	0.935
original_glszm_LargeAreaLowGrayLevelEmphasis	0.927	0.97	0.882	0.96	0.935
original_gldm_LargeDependenceEmphasis	0.907	0.852	0.956	0.993	0.927
original_glrlm_GrayLevelNonUniformityNormalized	0.98	0.959	0.85	0.908	0.924
original_glcm_Imc2	0.964	0.941	0.86	0.932	0.924
original_glrlm_RunPercentage	0.905	0.853	0.948	0.99	0.924
original_glcm_JointEnergy	0.977	0.957	0.807	0.933	0.919
original_firstorder_MeanAbsoluteDeviation	0.905	0.885	0.909	0.966	0.916
original_firstorder_Range	0.916	0.871	0.933	0.945	0.916
original_glcm_Autocorrelation	0.986	0.948	0.935	0.795	0.916
original_gldm_HighGrayLevelEmphasis	0.983	0.946	0.938	0.796	0.916
original_glrlm_LongRunHighGrayLevelEmphasis	0.97	0.963	0.919	0.808	0.915
original_glszm_LargeAreaEmphasis	0.926	0.97	0.765	0.998	0.915
original_glrlm_RunEntropy	0.906	0.885	0.895	0.959	0.911
original_shape_SurfaceVolumeRatio	0.816	0.908	0.974	0.948	0.911
original_ngtdm_Coarseness	0.989	0.938	0.813	0.897	0.909
original_glszm_GrayLevelNonUniformityNormalized	0.981	0.925	0.833	0.888	0.907
original_glrlm_HighGrayLevelRunEmphasis	0.967	0.925	0.938	0.796	0.907
original_glcm_SumAverage	0.986	0.952	0.922	0.767	0.907
original_glcm_JointAverage	0.986	0.952	0.922	0.767	0.907
original_glrlm_ShortRunHighGrayLevelEmphasis	0.938	0.905	0.945	0.832	0.905
original_glszm_HighGrayLevelZoneEmphasis	0.961	0.916	0.935	0.807	0.905
original_glcm_Idn	0.94	0.942	0.813	0.899	0.898
original_firstorder_RobustMeanAbsoluteDeviation	0.89	0.859	0.873	0.97	0.898
original_glrlm_ShortRunEmphasis	0.866	0.818	0.919	0.976	0.895
original_firstorder_InterquartileRange	0.897	0.855	0.855	0.969	0.894
original_glszm_LargeAreaHighGrayLevelEmphasis	0.926	0.97	0.718	0.959	0.893
original_glcm_InverseVariance	0.94	0.942	0.737	0.951	0.892
original_glszm_GrayLevelNonUniformity	0.795	0.814	0.959	1.0	0.892
original_glcm_Correlation	0.96	0.908	0.751	0.945	0.891
original_gldm_LargeDependenceHighGrayLevelEmphasis	0.976	0.923	0.903	0.751	0.888
original_gldm_DependenceEntropy	0.895	0.812	0.878	0.965	0.888
original_glszm_SmallAreaHighGrayLevelEmphasis	0.924	0.86	0.913	0.839	0.884
original_glcm_MCC	0.97	0.896	0.801	0.865	0.883
original_glcm_Imc1	0.925	0.875	0.811	0.919	0.883
original_ngtdm_Contrast	0.939	0.927	0.781	0.832	0.87
original_glszm_SizeZoneNonUniformity	0.761	0.739	0.975	0.999	0.868
original_glszm_ZoneVariance	0.805	0.881	0.756	0.998	0.86
original_gldm_SmallDependenceHighGrayLevelEmphasis	0.843	0.763	0.936	0.897	0.86
original_glcm_Idmn	0.94	0.942	0.723	0.831	0.859
original_glrlm_RunLengthNonUniformityNormalized	0.77	0.762	0.916	0.98	0.857
original_firstorder_10Percentile	0.776	0.722	0.964	0.953	0.854
original_glszm_LowGrayLevelZoneEmphasis	0.961	0.916	0.844	0.688	0.852
original_glcm_ClusterShade	0.89	0.881	0.701	0.931	0.851
original_glrlm_LongRunLowGrayLevelEmphasis	0.972	0.969	0.806	0.647	0.848
original_gldm_DependenceNonUniformityNormalized	0.914	0.801	0.714	0.925	0.839
original_glszm_SizeZoneNonUniformityNormalized	0.944	0.906	0.608	0.889	0.837
original_ngtdm_Strength	0.907	0.713	0.844	0.867	0.833
original_gldm_DependenceVariance	0.77	0.681	0.882	0.975	0.827
original_glszm_ZonePercentage	0.765	0.671	0.893	0.972	0.825
original_glrlm_LowGrayLevelRunEmphasis	0.967	0.925	0.779	0.606	0.819
original_firstorder_Minimum	0.816	0.725	0.961	0.763	0.816
original_gldm_LowGrayLevelEmphasis	0.983	0.946	0.744	0.556	0.807
original_firstorder_Skewness	0.823	0.781	0.696	0.923	0.806
original_gldm_LargeDependenceLowGrayLevelEmphasis	0.949	0.903	0.802	0.471	0.781
original_gldm_SmallDependenceEmphasis	0.684	0.606	0.816	0.956	0.766
original_glrlm_ShortRunLowGrayLevelEmphasis	0.788	0.73	0.778	0.68	0.744
original_glszm_SmallAreaEmphasis	0.851	0.757	0.518	0.811	0.734
original_firstorder_Kurtosis	0.635	0.622	0.68	0.89	0.707
original_ngtdm_Busyness	0.42	0.367	0.936	0.967	0.672
original_glszm_SmallAreaLowGrayLevelEmphasis	0.686	0.509	0.757	0.716	0.667
original_gldm_SmallDependenceLowGrayLevelEmphasis	0.524	0.478	0.79	0.778	0.642
original_shape_Elongation	0.666	0.484	0.553	0.646	0.587

Table 2. ICC values for each feature sorted by mean ICC.
